# Preexisting Somatic Mutations of Estrogen Receptor Alpha (*ESR1*) in Early-Stage Primary Breast Cancer

**DOI:** 10.1093/jncics/pkab028

**Published:** 2021-04-22

**Authors:** Malin Dahlgren, Anthony M George, Christian Brueffer, Sergii Gladchuk, Yilun Chen, Johan Vallon-Christersson, Cecilia Hegardt, Jari Häkkinen, Lisa Rydén, Martin Malmberg, Christer Larsson, Sofia K Gruvberger-Saal, Anna Ehinger, Niklas Loman, Åke Borg, Lao H Saal

**Affiliations:** 1 Division of Oncology, Department of Clinical Sciences Lund, Lund University, Lund, Sweden; 2 Lund University Cancer Center, Medicon Village, Lund, Sweden; 3 Department of Surgery, Skåne University Hospital, Lund, Sweden; 4 Department of Oncology, Skåne University Hospital, Lund, Sweden; 5 Division of Translational Cancer Research, Department of Laboratory Medicine, Lund University, Lund, Sweden; 6 Current affiliation: Center for Molecular Diagnostics, Skåne University Hospital, Lund, Sweden (SKG-S); 7 Department of Pathology, Skåne University Hospital, Lund, Sweden

## Abstract

**Background:**

More than three-quarters of primary breast cancers are positive for estrogen receptor alpha (ER; encoded by the gene *ESR1*), the most important factor for directing anti-estrogenic endocrine therapy (ET). Recently, mutations in *ESR1* were identified as acquired mechanisms of resistance to ET, found in 12% to 55% of metastatic breast cancers treated previously with ET.

**Methods:**

We analyzed 3217 population-based invasive primary (nonmetastatic) breast cancers (within the SCAN-B study, ClinicalTrials.gov NCT02306096), sampled from initial diagnosis prior to any treatment, for the presence of *ESR1* mutations using RNA sequencing. Mutations were verified by droplet digital polymerase chain reaction on tumor and normal DNA. Patient outcomes were analyzed using Kaplan-Meier estimation and a series of 2-factor Cox regression multivariable analyses.

**Results:**

We identified *ESR1* resistance mutations in 30 tumors (0.9%), of which 29 were ER positive (1.1%). In ET-treated disease, presence of *ESR1* mutation was associated with poor relapse-free survival and overall survival (2-sided log-rank test *P* < .001 and *P* = .008, respectively), with hazard ratios of 3.00 (95% confidence interval = 1.56 to 5.88) and 2.51 (95% confidence interval = 1.24 to 5.07), respectively, which remained statistically significant when adjusted for other prognostic factors.

**Conclusions:**

These population-based results indicate that *ESR1* mutations at diagnosis of primary breast cancer occur in about 1% of women and identify for the first time in the adjuvant setting that such preexisting mutations are associated to eventual resistance to standard hormone therapy. If replicated, tumor *ESR1* screening should be considered in ER-positive primary breast cancer, and for patients with mutated disease, ER degraders such as fulvestrant or other therapeutic options may be considered as more appropriate.

The estrogen receptor alpha (ER; encoded by the *ESR1* gene) has been known for decades as a targetable driver of breast tumor growth. Standard of care for ER-positive breast cancer includes endocrine therapy (ET), for example, treatment with estrogen receptor modulators such tamoxifen, aromatase inhibitors such as letrozole in the adjuvant and advanced settings, and ER degraders such as fulvestrant in the advanced setting. Recently, recurrent mutations in *ESR1* were identified in 12% to 55% of metastatic breast cancers, enriched among patients who had previously received ET (1-4). In these studies, a series of mutations were described, most of them located in the ligand-binding domain of the estrogen receptor, with the main hotspot among the amino acid residues 536-538 (3). Mutations in this site allow stabilization of the receptor in the more active, agonist conformation, leading to increased downstream transcription of ER targets. The endocrine-resistance mutations include at least 13 variants [reviewed in (5), also see Supplementary Table 1, available online] that have been experimentally verified to confer increased activity in the absence of estrogenic ligands, some of which have been associated to resistance to ET (1-4,6).

Depending on the amino acid substitution, functionally active ligand-binding domain mutations have also been shown to increase tumor cell growth and migration in monolayer cell culture (3,4,7) and xenograft growth in mouse models (1). Among the most commonly affected sites is amino acid Y537 with substitutions of S, C, D, or N along with D538G, all giving rise to increased ER activity (7,8). Although apparently similar with regard to mechanism, in vitro experiments show differing potency to confer ligand-independent and modulator-resistant growth, with Y537S being the most potent and others such as E380Q more moderate in its effects (8). Additionally, recent studies have revealed that different variants also give rise to distinct transcriptional phenotypes (9,10).

In contrast to the high rate of *ESR1* mutation in advanced breast cancer (3,4,6,11), the prevalence of resistance mutations in primary breast tumors has been reportedly very low, ranging from 0% to 7% in published studies (3,4,12-14). In cases of *ESR1* mutation-positive metastatic disease, the matched primary tumors when tested have been predominantly mutation negative, suggesting that many of these mutations are selected for under therapeutic pressure and during tumor progression (5,14).

In this study, we aimed to expand on the understanding of the ER-activating *ESR1* resistance mutations in primary breast cancer and investigate the relationship of preexisting *ESR1* mutations to ET resistance across a very large, real-world population-based early breast cancer cohort. The SCAN-B initiative (ClinicalTrials.gov NCT02306096) (15-17), initiated in 2010, is the largest prospective population-based collection of breast tumor samples undergoing routine RNA sequencing (RNA-seq); all newly diagnosed breast cancer patients in the participating 9 hospitals are offered enrollment. In the present study—the largest to our knowledge—we have analyzed the RNA-seq data of 3217 primary breast tumors for *ESR1* resistance mutations and, for the first time, identify the association of such mutations to clinical outcomes in the adjuvant treatment setting.

## Methods

### SCAN-B Cohort and RNA Sequencing

The study was approved by the Regional Ethical Review Board of Lund (diary numbers 2007/155, 2009/658, 2010/383, 2012/58, 2013/459) and the Swedish Data Inspection group (364-2010). Written informed consent is obtained from all study participants. The SCAN-B study is a multicenter prospective study that has enrolled more than 16 000 primary breast cancer patients to date and performs RNA sequencing on the tumor samples within days of surgery (15,16). RNA and DNA are isolated from tumor specimens using Qiagen AllPrep method (15,16). The 3217-patient SCAN-B series studied herein corresponds to an updated version of the patient group previously described by Brueffer et al. (17). Clinical parameters were determined as per the Swedish standard clinical routine, with greater than 10% positive cells by immunohistochemistry (IHC) scored as hormone receptor positive. Ki67 IHC status was established using local cutoffs (Swedish breast cancer quality assurance guidelines recommend that each laboratory calibrate a cutoff yearly such that one-third of 100 consecutive cases are Ki67 high). Grading was performed according to the Nottingham grading system (18) and pathological staging according to the TNM classification of malignant tumors (American Joint Committee on Cancer, 7th ed.). Updated clinical data and follow-up was retrieved from the Swedish National Quality Register.

### Mutations in RNA-Sequencing Data

For detailed protocols of RNA-sequencing data processing, including variant calling annotation, and filtering, see accompanying Supplementary Methods (available online) and Brueffer et al. (19). In brief, raw RNA-seq reads were trimmed and filtered as previously described (17). Reads were further processed using a modified version of the bcbio-nextgen 1.0.2 variant pipeline, aligned to the GRCh38.p8 reference genome using HISAT2 2.0.5, and duplicates marked using SAMBLASTER 0.1.24. Variants were called using VarDict-Java 1.5.0 requiring a minimum variant allele fraction of 2.0%. To eliminate germline calls and artifacts resulting from sources such as library preparation and RNA editing, the variants were annotated and filtered using a variety of data sources (Supplementary Methods, available online). Finally, filtered but likely true variant calls were rescued using databases such as the Catalogue of Somatic Mutations in Cancer. The resulting list of mutations was then searched for any of the 13 *ESR1* ET resistance mutations previously reported in the literature (Supplementary Table 1, available online).

### Droplet Digital PCR

IBSAFE assays were designed for 5 of the most recurrent *ESR1* variants: E380Q, D538G, Y537S, Y537N, and Y537C (SAGAsafe; SAGA Diagnostics AB, Lund, Sweden). Assays were validated using fragmented human male normal DNA (Thermo Fisher Scientific Inc, Waltham, MA, USA) as a negative control and gBlock synthetic DNA sequences (Integrated DNA Technologies BVBA, Leuven, Belgium), containing the respective mutant alleles spiked into wild-type DNA, as positive controls. IBSAFE droplet digital polymerase chain reaction (ddPCR) was performed as previously described (20). For validation of variants found by RNA-seq, 10 ng of genomic tumor DNA or germline DNA isolated from whole blood was used.

### Statistical Analyses

Data analysis, data visualization, and statistics were performed using R version 3.6.2 (2019-12-12) with relevant packages (see the Supplementary Methods, available online). For 2-group comparisons, Fisher exact test and Mann-Whitney U test were used. Overall survival (OS) events were defined as death from any cause and relapse-free survival (RFS) events as death from any cause or recurrence of breast cancer (locoregional or distant). *P* values for survival were calculated using the log-rank test. A series of 2-factor Cox regression multivariable analyses were performed adjusting for the following clinical variables: age at diagnosis (50 years and older or younger than 50 years old), tumor size (≥20 or <20 mm), lymph node status, tumor grade, and tumor pathological stage. The proportional hazards assumption was tested using Schoenfeld residuals. Absolute risk for outcome was calculated using the number of events divided by the number of patients with follow-up data in the given group. All tests were 2-sided, and *P* values of .05 or less were considered statistically significant.

## Results

### Clinical Characteristics

The population-based cohort of 3217 primary breast tumors was RNA sequenced within the framework of the SCAN-B study (15,16). The clinical characteristics of the cohort represent the expected distribution for early breast cancer in Sweden (Table 1): the median patient age at diagnosis was 64 years; 84.6% were ER positive; 71.7% progesterone receptor (PR) positive; 12.9% had HER2 amplification; 35.5% were positive for tumor spread to at least 1 lymph node at the time of surgery; 15.0% were Nottingham histological grade 1, 46.9% grade 2, and 36.1% grade 3. RNA-seq–based gene expression data was also used to determine the PAM50 molecular subtypes of the tumors: 48.0% were classified to the luminal A group, 27.9% in the luminal B group, 8.7% HER2-enriched, 9.9% basal-like, and 3.5% normal-like tumors. Postoperative endocrine treatment had been administered to 77.7% of the patients in the cohort, corresponding to 90.5% of the ER-positive subgroup. ET was given as sole adjuvant therapy to 1579 patients, whereas 914 patients also received chemotherapy. The median follow-up time after diagnosis was 6.29 years.

**Table 1.** Clinical parameters of the patient cohort and for the cases with *ESR1* endocrine therapy-resistance mutation

Clinical parameters	All cases (n = 3217)	ER-positive group (n = 2720)	Endocrine therapy (ET)-treated group (n = 2499)	ESR1 mutation-positive cases (n = 30)	*P*		
					ESR1 mutation vs wild-type in all cases	ESR1 mutation vs wild-type in ER positive group	ESR1 mutation vs wild-type in ET-treated group
Patient age^a^							
Median (range), y	64 (24-96)	64 (24-96)	64 (24-95)	64.5 (52-85)	.046^c^	.049^c^	.12^c^
< 50 years old, No. (%)	597 (18.6)	480 (17.6)	460 (18.4)	0 (0)	.004^d^	.006^d^	.01^d^
≥ 50 years old, No. (%)	2620 (81.4)	2240 (82.4)	2039 (81.6)	30 (100.0)			
Tumor size							
Median (range), mm	17 (1-126)	17 (1-126)	18 (1-126)	21 (8-50)	.14^c^	.10^c^	.08^c^
Lymph node status, No. (%)							
Node positive	1141 (35.5)	963 (35.4)	949 (38.0)	11 (36.7)	.70^d^	.69^d^	.69^d^
Node negative	1913 (59.5)	1629 (59.9)	1430 (57.2)	16 (53.3)			
NA	163 (5.1)	128 (4.7)	120 (4.8)	3 (10.0)			
Histological grade, No. (%)							
1	483 (15)	476 (17.5)	360 (14.4)	5 (16.7)	.75^d^	1.00^d^	1.00^d^
2	1509 (46.9)	1452 (53.4)	1346 (53.9)	16 (53.3)			
3	1161 (36.1)	757 (27.8)	751 (30.1)	9 (30.0)			
NA	64 (2.0)	35 (1.3)	42 (1.7)	0 (0)			
Estrogen receptor (ER), No. (%)							
ER positive	2720 (84.6)	2720 (100)	2462 (98.5)	29 (96.7)	.11^d^	1.00^d^	1.00^d^
ER negative	463 (14.4)	0 (0)	21 (0.8)	1 (3.3)			
NA	34 (1.1)	0 (0)	16 (0.6)	0 (0)			
Progesterone receptor (PR), No. (%)							
PR positive	2308 (71.7)	2287 (84.1)	2083 (83.4)	26 (86.7)	.14^d^	.79^d^	.41^d^
PR negative	824 (25.6)	375 (13.8)	357 (14.3)	4 (13.3)			
NA	85 (2.6)	58 (2.1)	59 (2.4)	0 (0)			
HER2 receptor, No. (%)							
HER2 positive	414 (12.9)	288 (10.6)	286 (11.4)	4 (13.3)	.79^d^	1.00^d^	1.00^d^
HER2 negative	2651 (82.4)	2312 (85.0)	2102 (84.1)	24 (80.0)			
NA	152 (4.7)	120 (4.4)	111 (4.4)	2 (6.7)			
Summary receptor status, No. (%)							
ER+ or PR+ and HER2+	294 (9.1)	288 (10.6)	279 (11.2)	3 (10.0)	.29^d^	1.00^d^	1.00^d^
ER+ or PR+ and HER2-	2327 (72.3)	2312 (85)	2092 (83.7)	24 (80.0)			
ER-/PR- and HER2+	112 (3.5)	0 (0)	3 (0.1)	1 (3.3)			
ER-/PR- and HER2-	310 (9.6)	0 (0)	5 (0.2)	0 (0)			
Ki67, No. (%)							
Ki67 high	887 (27.6)	677 (21.0)	654 (20.3)	4 (13.3)	.33^d^	.53^d^	.52^d^
Ki67 low	627 (19.5)	615 (19.1)	513 (15.9)	6 (20.0)			
Ki67 NA	1703 (52.9)	1428 (44.4)	1332 (41.4)	20 (66.7)			
PAM50 subtype,^b^ No. (%)							
Luminal A	1545 (48.0)	1519 (55.8)	1340 (53.6)	17 (56.7)	.23^d^	.76^d^	.74^d^
Luminal B	899 (27.9)	891 (32.8)	855 (34.2)	11 (36.7)			
HER2 enriched	279 (8.7)	125 (4.6)	132 (5.3)	0 (0)			
Basal-like	318 (9.9)	34 (1.3)	37 (1.5)	1 (3.3)			
Normal-like	112 (3.5)	90 (3.3)	78 (3.1)	0 (0)			
Unclassified	64 (2.0)	61 (2.2)	57 (2.3)	1 (3.3)			
Treatment received, No. (%)							
Endocrine treatment	2499 (77.7)	2462 (90.5)	2499 (100)	27 (90.0)	—	—	—
Tamoxifen	1317 (40.9)	1297 (47.7)	1317 (52.7)	12 (40.0)			
Aromatase inhibitor	1242 (38.6)	1226 (45.1)	1241 (49.7)	15 (50.0)			
Chemotherapy	1274 (39.6)	901 (33.1)	914 (36.6)	11 (36.7)			
HER2 therapy	348 (10.8)	232 (8.5)	238 (9.5)	2 (6.7)			
Radiotherapy	2058 (64.0)	1747 (64.2)	1585 (63.4)	20 (66.7)			
Systemic treatment regimen, No. (%)							
No systemic treatment	336 (10.4)	233 (8.6)	0 (0)	2 (6.7)	—	—	—
Endocrine treatment alone	1579 (49.1)	1571 (57.8)	1579 (63.2)	16 (53.3)			
Chemotherapy alone	252 (7.8)	11 (0.4)	0 (0)	0 (0)			
ET and chemotherapy	914 (28.4)	885 (32.5)	914 (36.6)	10 (33.3)			
Survival data							
Patients with OS data, No.	3199	2702	2483	30	—	—	—
Death events, No. (%)	467 (14.6)	351 (13.0)	320 (12.9)	8 (26.7)			
Patients with RFS data, No.	3058	2594	2391	30			
Relapse events, No. (%)	397 (13.0)	301 (11.6)	278 (11.6)	9 (30.0)			

a Clinicopathological information was retrieved from the Swedish National Quality Register for breast cancer via SCAN-B. Variables are defined as in the standard Swedish clinical routine, with Ki67 status determined using local cutoffs. ET = endocrine therapy; OS = overall survival; NA = not available; RFS = relapse-free survival; – = test not performed.

b PAM50 subtyping is derived from the RNA-sequencing data as described in Brueffer et al. (17).

c Two-sided Mann-Whitney U test *P* values.

d Two-sided Fisher exact test *P* values.

### Identification of Resistance Mutations

**Figure 1.**
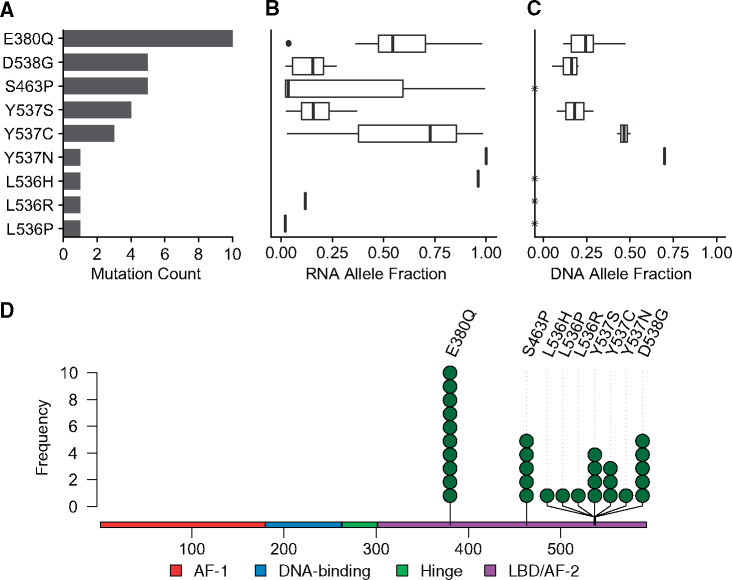
*ESR1* mutation screening at diagnosis in early breast cancer. 3217 tumors from the SCAN-B cohort were analyzed for mutations in the *ESR1* gene by RNA sequencing (RNA-seq). 31 *ESR1* resistance mutations were identified in 30 tumor samples (0.9%), with 1 double mutant tumor, harboring both D538G and Y537S variants. Shown are the count of each mutation (**A**), the mutation to wild-type allele fraction in RNA-seq data (**B**), and the mutant allele fraction in tumor DNA for 18 of 18 cases tested (8 E380Q, 4 D538G, and 1 each for Y537C/N/S) using IBSAFE ddPCR (**C**). Asterisk (*) indicates not tested. See **Supplementary Table 2** (available online) for additional details. (**D**) Lollipop plot for identified *ESR1* endocrine resistance mutations in the ER protein indicating the functional domains.

We searched available resources and literature to identify the *ESR1* mutations that have been previously shown to confer resistance to ET and identified 13 functionally validated mutations (Supplementary Table 1, available online). Mutation calling and annotation of the SCAN-B RNA-seq data identified 30 samples with an *ESR1* mutation known to cause endocrine resistance, corresponding to 0.9% of all tumors or 1.1% of the ER-positive tumors. The mutations that we identified included 10 tumors with E380Q, 5 with D538G, 5 with S463P, 4 with Y537S, 3 with Y537C, and 1 each of Y537N, L536H, L536P, and L536R at mutant allele fractions (MAFs) varying from 2.0% to 100% (Figure 1). One tumor harbored 2 resistance mutations, D538G and Y537S, at MAFs of 2.1% and 12.5%, respectively.

The *ESR1* ET resistance mutation group was similar to the patients with wild-type tumors in the ER-positive and ET-treated cohorts with respect to clinical factors and PAM50 molecular subtypes (Table 1). Patients with *ESR1* resistance mutations had a median age of diagnosis of 64.5 years vs 64 years for *ESR1* wild-type cases in both the full cohort, the ER-positive cohort, and the group receiving ET (Mann-Whitney U test, *P* = .046, *P* = .049, and *P* = .12, respectively; Table 1). Notably, 100% of the patients in the mutant group were 50 years or older, compared with 81% to 82% in wild-type groups (Mann-Whitney U test, *P* = .004, *P* = .006, and *P* = .01, respectively; Table 1). Of the 30 mutant cases, 29 were ER and/or PR positive, 3 of which were also HER2 amplified; 1 case was ER and PR negativeand HER2 amplified, and no mutant cases were triple negative. The percentage of ER positive cells determined by IHC was available for 22 of the *ESR1*-mutated tumors and ranged from 0% to 100% with a mean of 90.9%. Notably, none of the mutant tumors had been exposed to neoadjuvant endocrine therapy prior to the removal of the surgical specimens used for mutation screening. Postsurgery, 27 of the patients were given adjuvant treatment with tamoxifen or aromatase inhibitor, 16 of which received only ET, and 11 of which received chemotherapy in combination with ET; 2 patients received no systemic adjuvant therapy (Table 1). Detailed clinical parameters and therapies for the patients with *ESR1* mutations are provided in Supplementary Table 2 (available online).

### Validation of Variants

To validate the RNA-seq results using an orthogonal method, we used IBSAFE ddPCR mutation detection assays for the most recurrent amino acids affected (residues 380, 537, and 538) and retrieved tumor and blood DNA from the biobank for those samples that were available. In 100% (18 of 18), the mutations were validated in tumor DNA, and in 100% (11 of 11) with matching germline DNA from blood, the mutations were confirmed as somatic (Supplementary Table 3, available online). In tumor DNA, the MAFs varied from 5.2% to 70.0% and generally reflected the frequencies determined from RNA sequencing (Figure 1, C).

### *ESR1* Mutation and Patient Outcome

**Figure 2.**
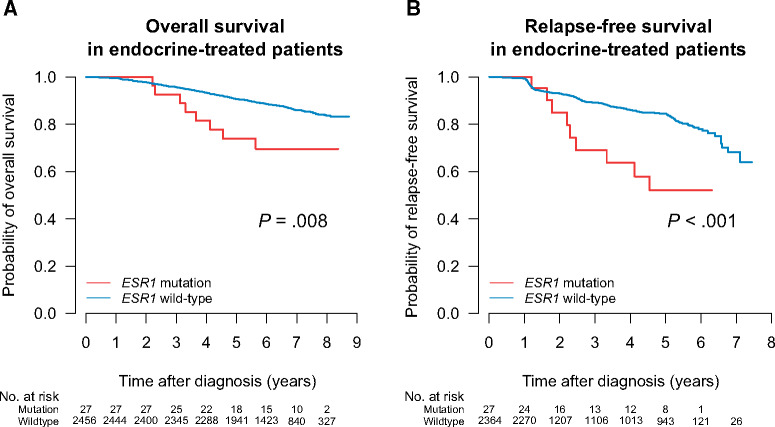
*ESR1* mutation at diagnosis of early breast cancer and outcome in patients who receive subsequent endocrine therapy. Kaplan-Meier survival plots for overall survival (**A**) and relapse-free survival (**B**) in the endocrine therapy group.

Based on evidence from metastatic breast cancer, we hypothesized that early-stage breast cancers with preexisting ET-resistance *ESR1* mutations would be poor responders to standard endocrine therapies. To test this, we evaluated patient survival using Kaplan-Meier analyses and log-rank tests of patients with or without 1 of the mutations of interest. Included in the ET group (n = 2499) were all patients that had undergone any type of adjuvant hormonal therapy. Of these patients, 914 also received chemotherapy as part of their regimen. We found that *ESR1* mutations had a negative effect on outcome in ET-treated tumors (Figure 2, A and B), both with regard to OS (*P* = .008, log-rank test) and RFS (*P* < .001). This association was also seen within the entire ER-positive patient cohort (Supplementary Figure 1, available online). The absolute risk for death in the endocrine-treated patients with *ESR1* wild-type was 12.7% (n = 2456, 312 events) compared with 29.6% (n = 27, 8 events) in the *ESR1* mutant group. Similarly, the absolute risk for an RFS event (death or relapse) was 11.4% (n = 2364, 269 events) for *ESR1* wild-type and 33.3% (n = 27, 9 events) for the mutant group. Four mutation-positive patients suffered a relapse to date; out of these, 2 were positive for D538G, 1 for Y537C, and 1 for Y537N. All 4 received adjuvant treatment with aromatase inhibitors (Supplementary Table 3, available online). To note, for 2 of these patients, we were able to obtain paired tissue samples from the relapse and ascertain *ESR1* mutation status in the metastatic site: case S002666 had confirmed Y537C mutation in the lung metastasis at a MAF of 81.9%, and case S005693 had confirmed Y537N mutation in the thoracic wall metastasis at a MAF of 62.5%.

**Figure 3.**
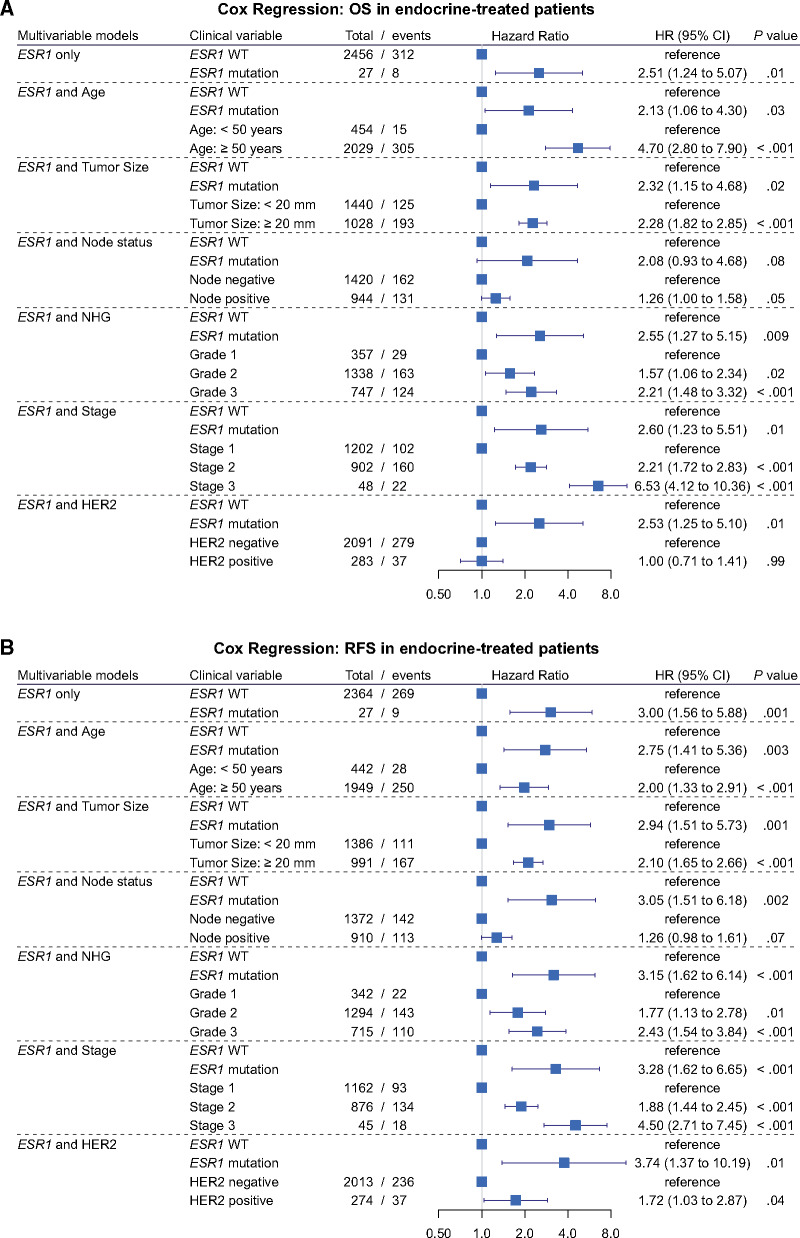
Cox regression models for *ESR1* mutation at diagnosis in early breast cancer. Forest plots for overall survival (**A**) and relapse-free survival (**B**) in the endocrine-treated group. HR = hazard ratio; OS = overall survival; RFS = relapse-free survival; WT = wild-type; NHG = Nottingham histological grade.

Because of the low total number of mutant cases and outcome events, we performed a series of 2-factor Cox regression multivariable analyses, adjusting in turn for the known prognostic variables age, tumor size, lymph node status, tumor grade, tumor pathological stage, or HER2 status (Figure 3). The unadjusted hazard ratio for OS was 2.51 (95% confidence interval [CI] = 1.24 to 5.07; *P* = .01) and ranged between 2.13 when considering age and 2.60 when considering stage, remaining a statistically significant variable in all analyses except when adjusted for lymph node status (Figure 3, A). Similarly, for RFS, *ESR1* mutation had an unadjusted hazard ratio of 3.00 (95% CI = 1.56 to 5.88; *P* = .001), with hazard ratios ranging from 2.75 to 3.74 in the multivariable models, remaining statistically significant in all Cox multivariable models (Figure 3, B).

## Discussion

To our knowledge, we have conducted the largest study of *ESR1* ET resistance mutations in treatment-naïve primary breast cancer. Using RNA sequencing of primary breast tumor samples taken at surgery and with a detection limit of minimum 2% MAF, the overall frequency of *ESR1* ET resistance mutations in the population-based SCAN-B cohort was 0.9%, corresponding to 1.1% among the ER-positive tumors. When linked to eventual therapies administered and patient outcomes, we show for the first time that preexisting *ESR1* mutation at diagnosis was statistically significantly associated to poor OS and RFS in the group that received adjuvant endocrine therapy with tamoxifen or aromatase inhibitor. A limitation of this study is the low number of mutant tumors and events, which did not allow for a Cox regression analysis adjusted for all prognostic factors in a single model. Adjustment for all prognostic factors will require a larger collaborative effort. However, in a series of pairwise-adjusted models, we show that resistance mutations at diagnosis of primary breast cancer were associated with a more than 2 times higher risk for death and 3 times higher risk for death or relapse.

There are several limitations to using RNA sequencing for mutation detection. The sequencing coverage of each gene is governed by its degree of expression, and therefore, highly transcribed genes will have a higher sequence coverage. Mutations in genes that are not sufficiently expressed or that lead to nonsense-mediated decay of the transcript can be missed, and subclonal mutations at low MAF less than 2% may not be detected. Moreover, RNA editing and artifacts from library preparation and sequencing have the potential to generate false-positive mutation calls. In the case of the ER, this is not a major issue because all mutations of interest are generally well-characterized missense activating mutations, and *ESR1* is generally highly expressed in the tumors, where ER is well established to be biologically relevant. It is possible that we may miss *ESR1* mutations in ER-negative tumors, although the biological meaning of such variants is unlikely to be pertinent in the adjuvant setting. The successful validation of the variants in tumor DNA for 18 of 18 mutations and not present in 11 of 11 matched germline DNA confirms that our RNA-seq–based approach is specific for detection of true-positive somatic *ESR1* mutations.

The Y537S, Y537N, Y537C, and D538G variants were found in 13 samples in our cohort. These altered ER proteins have previously been well characterized and shown to be powerful activators of downstream ER signaling. The most commonly occurring resistance mutation in our study, E380Q, is also the most common variant in primary breast tumors within The Cancer Genome Atlas (TCGA) (21). E380Q has been described as a gain-of-function variant that confers a growth advantage to cells in the absence of estrogen as well as relative resistance to tamoxifen and fulvestrant (6), although it may have a less potent effect on estrogen receptor activity in vitro compared with other mutations (8). E380Q has also been detected in other studies of treatment-resistant breast tumors (22-24), but its effects in vivo are not well understood. Of note, none of the E380Q-positive cases in our cohort suffered a relapse, and only 1 case had a death event.

Interestingly, all *ESR1* mutants occurred in patients who were diagnosed at age 50 years or older. Of these, patient surveys indicated that 96.7% were postmenopausal. The origin of preexisting *ESR1* mutation is unclear. One hypothesis is that they arise through age-related stochastic mutational events. Another hypothesis is that hormone replacement therapy could supply estrogenic signaling that may select for *ESR1*-mutant premalignant clones. Unfortunately, in this study, no data on hormone replacement therapy were available. These hypotheses should be investigated in future studies.

The presence of ET resistance mutations has been demonstrated to adversely affect OS by a median of almost 12 months in metastatic breast cancer (11). Here, we demonstrate for the first time that *ESR1* mutation is detrimental in primary breast cancer, showing that *ESR1* ET resistance mutations that preexist any systemic therapy are associated to poor OS and RFS in patients who receive adjuvant ET. These results contribute additional evidence that ER mutations impact the response to endocrine therapy. Mutant ER receptors can have diverse drug sensitivity profiles, and therefore, it is of great importance to enable detection and genotyping of these variants to aid clinicians in the decision process for choice of treatments. Presence of even a small population of ET-resistant cells could result in treatment failure and recurrence, and high-sensitivity screening using tissue and liquid biopsy plasma-circulating tumor DNA tests such as IBSAFE may be informative, not only for prognosis prediction but also for helping inform clinical decisions on which hormonal treatment options may most effectively reduce the risk of relapse for individual patients.

In conclusion, our results suggest that preexisting *ESR1* ET resistance mutations in untreated primary breast cancer are rare but are associated to poor outcome and resistance to standard hormone therapy. If our results are replicated, *ESR1* screening should be considered in ER-positive primary breast cancer, and for patients with mutated disease, ER degraders such as fulvestrant or other agents in development may be more appropriate.

## Funding

This work was supported by the Mrs Berta Kamprad Foundation and was also funded in part by the Swedish Foundation for Strategic Research; Swedish Research Council; Swedish Cancer Society; Knut and Alice Wallenberg Foundation; VINNOVA; Governmental Funding of Clinical Research within National Health Service; Lund University Medical Faculty; Gunnar Nilsson Cancer Foundation; Skåne University Hospital Foundation; BioCARE Research Program; King Gustav Vth Jubilee Foundation; Krapperup Foundation; Lund-Lausanne L2-Bridge/Biltema Foundation; and the Mats Paulsson Foundation.

## Notes

**Role of the funders:** The funders had no role in study design, collection, analysis, and interpretation of the data.

**Disclosures:** AMG, CB, YC, SG, and LHS have ownership interest (including stock, patents, etc.) in SAGA Diagnostics. LHS has received honoraria from Novartis and Boehringer-Ingelheim. The authors have declared no other potential conflicts of interest.

**Author contributions:** MD, SKG-S, and LHS conceived the study. MD, AMG, CB, SG, YC, JV-C, JH, NL, and LHS analyzed data. JV-C, CH, JH, LR, MM, CL, AE, NL, ÅB, and LHS established the SCAN-B initiative. CB, SG, and LHS established the RNA-seq mutation calling pipeline and filters. JV-C, CH, JH, LR, MM, CL, AE, NL, ÅB, and LHS provided clinical information. LHS supervised the project, and MD and LHS wrote the report with assistance from all authors. All authors discussed, critically revised, and approved the final version of the report for publication.

**Acknowledgements:** The authors thank the patients, clinicians, and hospital staff participating in the SCAN-B study, the staff at the central SCAN-B laboratory at Lund University, the Swedish National Breast Cancer Quality Registry (NKBC), Regional Cancer Center South, the South Swedish Breast Cancer Group (SSBCG), and the members of the Translational Oncogenomics Unit, Division of Oncology, Lund University.

**Prior presentations:** The work herein was presented orally at the American Association for Cancer Research (AACR) Annual Meeting in April 2020.

## Data Availability

The RNA-sequencing data is publicly available from the NCBI Gene Expression Omnibus database, accession GSE81540 (https://www.ncbi.nlm.nih.gov/geo/query/acc.cgi?acc=GSE81540).

## Supplementary Material

pkab028_Supplementary_Data
