# Supplementary material for: Preexisting Somatic Mutations of Estrogen Receptor Alpha (ESR1) in Early-Stage Primary Breast Cancer
Source: JNCI Cancer Spectr. 2021 Apr 22;5(2):pkab028. doi: 10.1093/jncics/pkab028 (PMC8060794; doi:10.1093/jncics/pkab028)
Supplement: pkab028_Supplementary_Data [file pkab028_supplementary_data.pdf]

Pre-existing Somatic Mutations of Estrogen Receptor Alpha (*ESR1*) in Early-stage Primary Breast Cancer

Malin Dahlgren[1,2], MSc, Anthony M. George[1,2], MSc, Christian Brueffer[1,2], PhD, Sergii Gladchuk[1,2], MSc, Yilun Chen[1,2], MSc, Johan Vallon-Christersson[1,2], PhD, Cecilia Hegardt[1,2], PhD, Jari Häkkinen[1,2], PhD, Lisa Rydén[3], MD, PhD, Martin Malmberg[4], MD, PhD, Christer Larsson[5], PhD, Sofia K. Gruvberger-Saal[1,†], PhD, Anna Ehinger[6], MD, PhD, Niklas Loman[4], MD, PhD, Åke Borg[1,2], PhD, Lao H. Saal[1,2,*], MD, PhD

[1] Division of Oncology, Department of Clinical Sciences Lund, Lund University, Lund, Sweden

[2] Lund University Cancer Center, Medicon Village, Lund, Sweden

[3] Department of Surgery, Skåne University Hospital, Lund, Sweden

[4] Department of Oncology, Skåne University Hospital, Lund, Sweden

[5] Division of Translational Cancer Research, Department of Laboratory Medicine, Lund University, Lund, Sweden

[6] Department of Pathology, Skåne University Hospital, Lund, Sweden

[†] Present address: Center for Molecular Diagnostics, Skåne University Hospital, Lund, Sweden

[*] To whom correspondence should be addressed: Berta Kamprad Associate Professor Lao H. Saal (lao.saal@med.lu.se; Twitter @LaoSaal), Division of Oncology, Department of Clinical Sciences Lund, Lund University Cancer Center, Medicon Village 404-B2, Lund University, SE-22381 Lund, Sweden. Tel: +46-46-2220365. Fax: +46-46-147327.

Supplementary Methods

Mutations in RNA-sequencing data

Processing of breast tumor RNA sequencing (RNA-seq) data and mutation calling was performed as described by Brueffer *et al* [1]. Raw reads were trimmed and filtered as described for the SCAN-B data analysis pipeline [2, 3]. Processing was then performed using a modified version of the bcbio-nextgen 1.0.2 variant pipeline (https://github.com/bcbio/bcbio-nextgen, modified version https://github.com/cbrueffer/bcbio-nextgen/tree/v1.0.2-scanb-calling). Reads were aligned using to the GRCh38.p8 reference genome (including alternative sequences and decoys), patched with dbSNP Build 147 common SNPs and the GENCODE 25 transcriptome model using HISAT2 2.0.5 [4] (with default options except --rna-strandness RF --rg-id ${ID_NAME} --rg PL:illumina --rg PU:${UNIT} --rg SM:${SAMPLE}). BAM index files were created using Sambamba 0.6.6 [5]. Duplicate reads were marked using SAMBLASTER 0.1.24 [6]. Somatic variants were called using VarDict-Java 1.5.0 [7] (with default options except -f 0.02 -N ${SAMPLE} -b ${BAM_FILE} -c 1 -S 2 -E 3 -g 4 -Q 10 -r 2 -q 20).

The mutations were then annotated using vcfanno 0.3.1 [8] with multiple resources. The databases used included dbSNP Build 151 [9], UCSC low complexity regions and Genome Aggregation Database (gnomAD) [10]. RNA-editing databases were RADAR v2 [11], DARNED [12], Malmö RNA-editing predicted sites [13] and RNA A-to-I editing database [14]. Annotation for variants with clinical relevance was done using CIViC [15], COSMIC [16, 17], MyCancerGenome [18], the Drug-Gene interaction database [19], and IntOGen-mutations [20]. Variants were also annotated for presence in resources for normal variants: Swegen database [21], Danish Genome project population reference [22], and in a manually constructed database containing variants from 10 normal tissue samples run through the SCAN-B pipeline. Variant effects were predicted using SnpEff 4.3.1r [23] with default parameters except hg38 -t -canon.

The variant filters were developed using another set of 275 tumors with matched normal DNA and available sequencing data both from DNA exons and RNA. Negative filters were applied for e.g. low complexity regions, SNP status and RNA editing. Positive filters were then applied to “rescue” true variants if present in databases for clinically relevant mutations in cancer, e.g. My Cancer Genome and COSMIC. The optimized variant filters were applied to

the 3217 tumor cohort mutation calls and the filtered variant list was interrogated for the presence of any of the 13 experimentally verified *ESR1* endocrine therapy resistance mutations.

Plots and statistics

Data analysis and statistics were performed using R version 3.6.2 (2019-12-12) and the packages stringr [24], survival [25], Hmisc [26], dplyr [27] and reshape2 [28]. Mutations were visualized using the trackviewer package [29] and RColorBrewer [30] as well as ggplot2 [31]. Overall survival endpoint events were defined as death from any cause. Relapse-free survival comprised death from any cause or the occurrence of local or distant metastasis.

Cox regression multivariable analysis was performed using survminer package [32]. Due to the low number of mutant tumors, we performed a series of two-factor multivariable analyses, adjusting for the following clinical variables: age at diagnosis (≥50 or <50 years old), tumor size (≥20 or <20 mm), lymph node status (positive or negative), tumor grade (1, 2, or 3), and tumor stage (1, 2, or 3).

Primers and probes

IBSAFE primers and probes (SAGAsafe; SAGA Diagnostics AB) were designed for five of the variants: E380Q, D538G, Y537S, Y537N and Y537C. Probes were designed for the wildtype *ESR1* variant (labeled with hexachloro-fluorescein; HEX) and one for each of the mutations (labeled with 6-fluorescein amidite; FAM). Primers and probes were validated using fragmented human male normal DNA (Thermo Fisher Scientific Inc.) as a negative control and gBlock synthetic DNA sequences (Integrated DNA Technologies BVBA), containing the respective mutant alleles spiked into the wildtype DNA, as positive control.

Droplet digital PCR

Droplet digital PCR (ddPCR) was performed using the Bio-Rad Supermix for ddPCR without dUTP. Each IBSAFE assay (SAGAsafe, SAGA Diagnostics AB) was performed in multiplex with one wildtype HEX-labeled probe, paired with mutant FAM-labeled probes. For validation of variants found with RNA-sequencing, 10 ng of genomic tumor DNA or germline DNA prepared from blood was analyzed.

Droplets were generated using a QX100 droplet generator (Bio-Rad) and subsequently run through PCR cycles on a C1000 Touch Thermal Cycler (Bio-Rad). Droplet fluorescence

amplitude for two channels (HEX for wildtype and FAM for mutant) was then measured on a QX200 Droplet Reader (Bio-Rad). Droplet fluorescence was read in both channels and indicated whether each droplet was: 1) positive for one of the mutant variants, 2) positive for the wildtype variant, 3) positive for both mutant and wildtype variants, or 4) negative for all of the assayed sequences, mutant and wildtype.

Droplet fluorescence amplitude data was processed in R. The absolute quantities of mutant and wildtype copies per microliter reaction were calculated taking into account the Poisson distribution using the formula: $C = -\ln(N)/V$, where C is the number of copies in the reaction, N is the fraction of negative droplets, V is the droplet volume (reported by Bio-Rad to be 0.85 nL). The allele frequency was then determined by dividing the number of mutant copies with the total number of allele copies.

References

1. Brueffer C, Gladchuk S, Winter C, et al. The mutational landscape of the SCAN-B real-world primary breast cancer transcriptome. *EMBO Mol Med* 2020;12(10):e12118.
2. Hakkinen J, Nordborg N, Mansson O, et al. Implementation of an Open Source Software solution for Laboratory Information Management and automated RNAseq data analysis in a large-scale Cancer Genomics initiative using BASE with extension package Reggie. *bioRxiv* 2016; 10.1101/038976.
3. Saal LH, Vallon-Christersson J, Hakkinen J, et al. The Sweden Cancerome Analysis Network - Breast (SCAN-B) Initiative: a large-scale multicenter infrastructure towards implementation of breast cancer genomic analyses in the clinical routine. *Genome Med* 2015;7(1):20.
4. Kim D, Langmead B, Salzberg SL. HISAT: a fast spliced aligner with low memory requirements. *Nat Methods* 2015;12(4):357-60.
5. Tarasov A, Vilella AJ, Cuppen E, et al. Sambamba: fast processing of NGS alignment formats. *Bioinformatics* 2015;31(12):2032-4.
6. Faust GG, Hall IM. SAMBLASTER: fast duplicate marking and structural variant read extraction. *Bioinformatics* 2014;30(17):2503-5.
7. Lai Z, Markovets A, Ahdesmaki M, et al. VarDict: a novel and versatile variant caller for next-generation sequencing in cancer research. *Nucleic Acids Res* 2016;44(11):e108.

8. Pedersen BS, Layer RM, Quinlan AR. Vcfanno: fast, flexible annotation of genetic variants. *Genome Biol* 2016;17(1):118.
9. Sherry ST, Ward MH, Kholodov M, et al. dbSNP: the NCBI database of genetic variation. *Nucleic Acids Res* 2001;29(1):308-11.
10. Karczewski KJ, Francioli LC, Tiao G, et al. Variation across 141,456 human exomes and genomes reveals the spectrum of loss-of-function intolerance across human protein-coding genes. *bioRxiv* 2019; 10.1101/531210:531210.
11. Ramaswami G, Li JB. RADAR: a rigorously annotated database of A-to-I RNA editing. *Nucleic Acids Res* 2014;42(Database issue):D109-13.
12. Kiran A, Baranov PV. DARNED: a DAtabase of RNa EDiting in humans. *Bioinformatics* 2010;26(14):1772-6.
13. Sun J, De Marinis Y, Osmark P, et al. Discriminative Prediction of A-To-I RNA Editing Events from DNA Sequence. *PLoS ONE* 2016;11(10):e0164962.
14. Picardi E, D'Erchia AM, Lo Giudice C, et al. REDIportal: a comprehensive database of A-to-I RNA editing events in humans. *Nucleic Acids Res* 2017;45(D1):D750-d757.
15. Griffith M, Spies NC, Krysiak K, et al. CIViC is a community knowledgebase for expert crowdsourcing the clinical interpretation of variants in cancer. *Nat Genet* 2017;49(2):170-174.
16. Forbes SA, Beare D, Boutselakis H, et al. COSMIC: somatic cancer genetics at high-resolution. *Nucleic Acids Res* 2017;45(D1):D777-D783.
17. Sondka Z, Bamford S, Cole CG, et al. The COSMIC Cancer Gene Census: describing genetic dysfunction across all human cancers. *Nat Rev Cancer* 2018;18(11):696-705.
18. Vanderbilt-Ingram Cancer Center. My Cancer Genome (release March 2016). http://mycancergenome.org.
19. Cotto KC, Wagner AH, Feng YY, et al. DGIdb 3.0: a redesign and expansion of the drug-gene interaction database. *Nucleic Acids Res* 2018;46(D1):D1068-D1073.
20. Gonzalez-Perez A, Perez-Llamas C, Deu-Pons J, et al. IntOGen-mutations identifies cancer drivers across tumor types. *Nat Methods* 2013;10(11):1081-2.
21. Ameur A, Dahlberg J, Olason P, et al. SweGen: a whole-genome data resource of genetic variability in a cross-section of the Swedish population. European Journal of *Human Genet* 2017;25(11):1253-1260.
22. Maretty L, Jensen JM, Petersen B, et al. Sequencing and de novo assembly of 150 genomes from Denmark as a population reference. *Nature* 2017;548(7665):87-91.

23. Cingolani P, Platts A, Wang le L, et al. A program for annotating and predicting the effects of single nucleotide polymorphisms, SnpEff: SNPs in the genome of Drosophila melanogaster strain w1118; iso-2; iso-3. *Fly* (Austin) 2012;6(2):80-92.
24. Wickham H. stringr: Simple, Consistent Wrappers for Common String Operations. https://CRAN.R-project.org/package=stringr.
25. Therneau T. A Package for Survival Analysis in R. https://CRAN.R-project.org/package=survival.
26. Harrell Jr FE. Hmisc: Harrell Miscellaneous. https://CRAN.R-project.org/package=Hmisc.
27. Wickham HF, Romain; Müller, Kirill. dplyr: A Grammar of Data Manipulation. https://CRAN.R-project.org/package=dplyr.
28. Wickham H. Reshaping Data with the {reshape} Package. *J Stat Softw* 2007;21(12):1-20.
29. Ou J, Zhu LJ. trackViewer: a Bioconductor package for interactive and integrative visualization of multi-omics data. *Nat Methods* 2019;16(6):453-454.
30. Neuwirth E. RColorBrewer: ColorBrewer Palettes. https://CRAN.R-project.org/package=RColorBrewer.
31. Wickham H. ggplot2: Elegant Graphics for Data Analysis. http://ggplot2.org.
32. Kassambara AK, Marcin; Biecek, Przemyslav. survminer: Drawing Survival Curves using 'ggplot2'. https://CRAN.R-project.org/package=survminer.

Supplementary Table 1. List of experimentally-validated *ESR1* endocrine therapy-resistance mutations and literature references.

Mutation	Nucleotide change	COSMIC ID	References
E380Q	c.1138G>C	COSM3829320	1-6
V422del	c.1262_1264delTGG	COSM1074628	4, 6
S463P	c.1387T>C	COSM4771561	2, 6, 7
L469V	c.1405C>G	-	6
L536H	c.1607T>A	COSM6201639	6, 7
L536Q	c.1607_1608TC>AG	COSM4766050	6
L536P	c.1607T>C	COSM6906109	6, 8
L536R	c.1607T>G	COSM4774826	2, 6
Y537C	c.1610A>G	COSM5413590	4-7, 9, 10
Y537D	c.1609T>G	COSM3680810	6
Y537N	c.1609T>A	COSM1074635	2, 6, 7, 9, 10
Y537S	c.1610A>C	COSM1074639	1-4, 6-10
D538G	c.1613A>G	COSM94250	2-11

References:

[1] Li S, Shen D, Shao J, Crowder R, Liu W, Prat A, et al. Endocrine-therapy-resistant ESR1 variants revealed by genomic characterization of breast-cancer-derived xenografts. Cell Rep. 2013;4:1116-30.

[2] Toy W, Shen Y, Won H, Green B, Sakr RA, Will M, et al. ESR1 ligand-binding domain mutations in hormone-resistant breast cancer. Nat Genet. 2013;45:1439-45.

[3] Guttery DS, Page K, Hills A, Woodley L, Marchese SD, Rghebi B, et al. Noninvasive detection of activating estrogen receptor 1 (ESR1) mutations in estrogen receptor-positive metastatic breast cancer. Clin Chem. 2015;61:974-82.

[4] Lefebvre C, Bachelot T, Filleron T, Pedrero M, Campone M, Soria JC, et al. Mutational Profile of Metastatic Breast Cancers: A Retrospective Analysis. PLoS Med. 2016;13:e1002201.

[5] Shaw JA, Guttery DS, Hills A, Fernandez-Garcia D, Page K, Rosales BM, et al. Mutation Analysis of Cell-Free DNA and Single Circulating Tumor Cells in Metastatic Breast Cancer Patients with High Circulating Tumor Cell Counts. Clin Cancer Res. 2017;23:88-96.

[6] Toy W, Weir H, Razavi P, Lawson M, Goeppert AU, Mazzola AM, et al. Activating ESR1 Mutations Differentially Affect the Efficacy of ER Antagonists. Cancer Discov. 2017;7:277-87.

[7] Yanagawa T, Kagara N, Miyake T, Tanei T, Naoi Y, Shimoda M, et al. Detection of ESR1 mutations in plasma and tumors from metastatic breast cancer patients using next-generation sequencing. Breast Cancer Res Treat. 2017;163:231-40.

[8] Yu M, Bardia A, Aceto N, Bersani F, Madden MW, Donaldson MC, et al. Cancer therapy. Ex vivo culture of circulating breast tumor cells for individualized testing of drug susceptibility. Science. 2014;345:216-20.

[9] Jeselsohn R, Yelensky R, Buchwalter G, Frampton G, Meric-Bernstam F, Gonzalez-Angulo AM, et al. Emergence of constitutively active estrogen receptor-alpha mutations in pretreated advanced estrogen receptor-positive breast cancer. Clin Cancer Res. 2014;20:1757-67.

[10] Robinson DR, Wu YM, Vats P, Su F, Lonigro RJ, Cao X, et al. Activating ESR1 mutations in hormone-resistant metastatic breast cancer. Nat Genet. 2013;45:1446-51.

[11] Merenbakh-Lamin K, Ben-Baruch N, Yeheskel A, Dvir A, Soussan-Gutman L, Jeselsohn R, et al. D538G mutation in estrogen receptor-alpha: A novel mechanism for acquired endocrine resistance in breast cancer. Cancer Res. 2013;73:6856-64.

Supplementary Table 2. Detailed clinical information for each patient with an ESR1 ET-resistance mutation.

Sample ID	Mutation	Age at diagnosis	Tumor size (mm)	T	N	M	Tumor stage	Lymph node status	Type	ER status	ER percent positive cells	PgR status	PgR percent positive cells	HER2 status	NHG	Endocrine therapy	Tamoxifen	Aromatase inhibitor	Chemo-therapy	Anti-HER2 therapy	Radio-therapy	Reported recurrence	Recurrence in	OS years	OS event	RFS years	RFS event
S000272	L536H	82	22	T1	N0	M0	Stage 1A	Negative	Ductal	Positive	NA	Positive	NA	Negative	G2	Yes	Yes							4.12	1	4.12	1
S000288	Y537S	61	12	T1	N0	M0	Stage 1A	Negative	Lobular	Positive	100	Positive	100	Negative	G2	Yes		Yes			Yes			8.38	0	6.31	0
S000326	S463P	85	27	T2	N0	M0	Stage 2A	Negative	Ductal	Positive	NA	Positive	NA	Positive	G3	Yes	Yes							2.21	1	2.21	1
S000394	E380Q	55	18	T1	N0	M0	Stage 1A	Negative	Ductal	Positive	NA	Positive	NA	Negative	G2	Yes	Yes				Yes			7.10	0	5.63	0
S000433	S463P	78	9	T0	N0	M0	NA	Negative	Ductal	Positive	100	Negative	100	Negative	G1						Yes			8.26	0	5.20	0
S000474	D538G	55	15	T1	N0	M0	Stage 1A	Negative	Ductal	Positive	100	Negative	100	Positive	G2	Yes	Yes				Yes			8.18	0	5.17	0
S000801	D538G	84	32	T2	N0	M0	Stage 2A	NA	Ductal	Positive	75	Positive	75	Negative	G2	Yes	Yes							4.55	1	4.55	1
S000871	D538G	81	25	T2	N0	M0	Stage 2A	Negative	Other	Positive	95	Positive	95	Negative	G3	Yes		Yes				Yes	Liver, lung, bone	3.66	1	1.78	1
S000949	D538G, Y537S	52	15	T1	N0	M0	Stage 1A	Negative	Ductal	Positive	NA	Negative	NA	Negative	G3	Yes	Yes		Yes					7.72	0	4.57	0
S001097	E380Q	65	15	T1	N0	M0	Stage 1A	Negative	Ductal	Positive	90	Positive	90	Negative	G2	Yes	Yes							7.58	0	5.12	0
S001160	E380Q	65	42	T2	N1	M0	Stage 2B	Positive	Ductal	Positive	85	Positive	85	Negative	G3	Yes		Yes	Yes		Yes			7.55	0	1.01	0
S001238	E380Q	64	27	T1	N0	M0	Stage 1A	Negative	Lobular	Positive	NA	Positive	NA	Negative	G2	Yes		Yes			Yes			7.51	0	5.22	0
S001265	S463P	79	45	T2	N1	M0	Stage 2B	Positive	Ductal	Positive	NA	Positive	NA	Negative	G3	Yes	Yes							2.29	1	2.29	1
S001445	D538G	74	34	T2	N0	M0	Stage 2A	Positive	Lobular	Positive	95	Positive	95	Negative	G3	Yes		Yes	Yes		Yes	Yes	Bone, axilla	7.30	0	3.33	1
S001632	L536R	64	11	T0	N0	M0	NA	Negative	Ductal	Positive	NA	Positive	NA	Negative	G1	Yes	Yes							7.15	0	5.31	0
S002195	Y537S	58	13	T1	N0	M0	Stage 1A	Negative	Ductal	Positive	NA	Positive	NA	Negative	G1	Yes	Yes				Yes			7.04	0	5.03	0
S002503	E380Q	54	11	T0	N0	M0	NA	Positive	Ductal	Positive	100	Positive	100	Negative	G1	Yes		Yes			Yes			6.70	0	5.11	0
S002666	Y537C	56	21	T4	N1	M0	Stage 3B	Positive	Ductal	Positive	95	Positive	95	Negative	G2	Yes		Yes	Yes		Yes	Yes	Lung	3.30	1	2.46	1
S002727	S463P	50	20	T0	N0	M0	NA	Negative	Ductal	Positive	100	Positive	100	Negative	G3	Yes	Yes		Yes		Yes			6.58	0	1.72	0
S003025	Y537C	64	23	T1	N0	M0	Stage 1A	Positive	Ductal	Positive	90	Positive	90	Negative	G2	Yes		Yes	Yes		Yes			6.34	0	0.99	0
S003561	Y537S	82	33	T2	N0	M0	Stage 2A	NA	Ductal	Positive	95	Positive	95	Negative	G2	Yes	Yes							5.64	1	0.98	0
S003655	L536P	84	13	T1	N0	M0	Stage 1A	Positive	Ductal	Positive	100	Positive	100	Negative	G1	Yes		Yes			Yes			6.07	0	0.38	0
S003656	E380Q	72	15	T1	N0	M0	Stage 1A	Positive	Ductal	Positive	100	Positive	100	Negative	G2	Yes		Yes	Yes		Yes			6.04	0	1.02	0
S003786	E380Q	84	30	T2	N1	M0	Stage 2B	Positive	Ductal	Positive	90	Positive	90	Positive	G3	Yes		Yes		Yes	Yes			5.92	0	4.03	0
S004578	E380Q	61	21	T1	N0	M0	Stage 1A	Negative	Ductal	Positive	95	Positive	95	Negative	G2	Yes		Yes	Yes		Yes			5.58	0	1.30	0
S005145	E380Q	63	15	T0	N0	M0	NA	Negative	Ductal	Positive	100	Positive	100	Negative	G2	Yes		Yes			Yes			3.12	1	1.20	1
S005147	E380Q	61	NA	T2	N1	M0	Stage 2B	NA	Ductal	Negative	0	Negative	0	Positive	G3				Yes	Yes	Yes			5.17	0	1.05	0
S005693	Y537N	69	21	T2	N0	M0	Stage 2A	Positive	Ductal	Positive	95	Positive	95	Negative	G2	Yes		Yes	Yes			Yes	Breast	4.93	0	1.63	1
S006576	Y537C	61	50	T2	N0	M0	Stage 2A	Positive	Lobular	Positive	100	Positive	100	NA	G2	Yes		Yes	Yes		Yes			4.39	0	1.08	0
S006733	S463P	84	8	T1	N0	M0	Stage 1A	Negative	Lobular	Positive	100	Positive	100	NA	G2						Yes			4.32	0	1.08	0

Supplementary Table 3. Validation results for 18 cases with tumor DNA and 11 cases with matching germline DNA.

Specimen	Amino acid change	Chromosome	Position	Nucleotide change	AF by RNA-seq (tumor RNA)	DNA testing of tumor and germline	AF in ddPCR (tumor DNA)	Somatic or germline
S000474	D538G	chr6	152098791	A>G	27.0%	tumor only	20.1%	NA
S000801	D538G	chr6	152098791	A>G	5.7%	tumor and germline	5.2%	somatic
S000871	D538G	chr6	152098791	A>G	15.4%	tumor and germline	19.1%	somatic
S001445	D538G	chr6	152098791	A>G	20.6%	tumor and germline	13.5%	somatic
S000394	E380Q	chr6	152011697	G>C	71.7%	tumor and germline	29.6%	somatic
S001097	E380Q	chr6	152011697	G>C	51.9%	tumor and germline	28.6%	somatic
S001160	E380Q	chr6	152011697	G>C	48.8%	tumor and germline	16.9%	somatic
S001238	E380Q	chr6	152011697	G>C	84.2%	tumor only	13.4%	NA
S002503	E380Q	chr6	152011697	G>C	36.4%	tumor and germline	11.6%	somatic
S003786	E380Q	chr6	152011697	G>C	47.1%	tumor only	28.2%	NA
S004578	E380Q	chr6	152011697	G>C	56.9%	tumor and germline	20.2%	somatic
S005145	E380Q	chr6	152011697	G>C	98.1%	tumor only	47.3%	NA
S002666	Y537C	chr6	152098788	A>G	98.4%	tumor and germline	50.2%	somatic
S006576	Y537C	chr6	152098788	A>G	72.6%	tumor only	42.7%	NA
S005693	Y537N	chr6	152098787	T>A	100.0%	tumor only	70.0%	NA
S000288	Y537S	chr6	152098788	A>C	18.9%	tumor and germline	18.0%	somatic
S000949	Y537S	chr6	152098788	A>C	12.5%	tumor and germline	7.6%	somatic
S003561	Y537S	chr6	152098788	A>C	37.1%	tumor only	28.9%	NA
